# Clinical and Pathological Aspects of Silent Pituitary Adenomas

**DOI:** 10.1210/jc.2018-00688

**Published:** 2018-07-17

**Authors:** Juliana Drummond, Federico Roncaroli, Ashley B Grossman, Márta Korbonits

**Affiliations:** 1 Centre for Endocrinology, William Harvey Research Institute, Barts and the London School of Medicine and Dentistry, Queen Mary University of London, London, United Kingdom; 2 Division of Neuroscience and Experimental Psychology, Faculty of Biology, Medicine and Health, University of Manchester, Manchester, United Kingdom

## Abstract

**Context:**

Silent pituitary adenomas are anterior pituitary tumors with hormone synthesis but without signs or symptoms of hormone hypersecretion. They have been increasingly recognized and represent challenging diagnostic issues.

**Evidence Acquisition:**

A comprehensive literature search was performed using MEDLINE and EMBASE databases from January 2000 to March 2018 with the following key words: (i) pituitary adenoma/tumor and nonfunctioning; or (ii) pituitary adenoma/tumor and silent. All titles and abstracts of the retrieved articles were reviewed, and recent advances in the field of silent pituitary adenomas were summarized.

**Evidence Synthesis:**

The clinical and biochemical picture of pituitary adenomas reflects a continuum between functional and silent adenomas. Although some adenomas are truly silent, others will show some evidence of biochemical hypersecretion or could have subtle clinical signs and, therefore, can be referred to as clinically silent or “whispering” adenomas. Silent tumors seem to be more aggressive than their secreting counterparts, with a greater recurrence rate. Transcription factors for pituitary cell lineages have been introduced into the 2017 World Health Organization guidelines: steroidogenic factor 1 staining for gonadotroph lineage; PIT1 (pituitary-specific positive transcription factor 1) for growth hormone, prolactin, and TSH lineage, and TPIT for the corticotroph lineage. Prospective studies applying these criteria will establish the value of the new classification.

**Conclusions:**

A concise review of the clinical and pathological aspects of silent pituitary adenomas was conducted in view of the new World Health Organization classification of pituitary adenomas. New classifications, novel prognostics markers, and emerging imaging and therapeutic approaches need to be evaluated to better serve this unique group of patients.

## Teaser

We present a concise review of the clinical and pathological aspects of silent pituitary adenomas in view of the new World Health Organization classification of pituitary adenomas.

**Figure 1.**
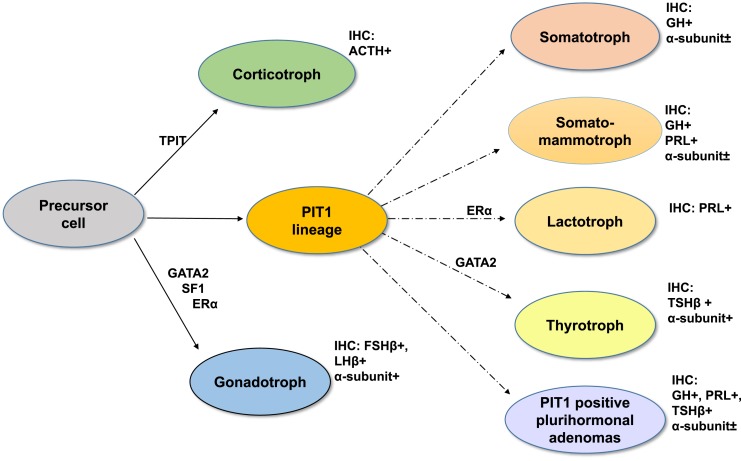
During pituitary development, specific transcription factors are fundamental to the complex process of adenohypophyseal cell differentiation. The three main pathways of cell differentiation and the immunoprofile of each cell lineage are illustrated. GATA2, GATA binding protein-2; PROP1, PROP paired-like homeobox 1 (also called prophet of PIT1); PIT1, POU class 1 homeobox 1 (PUO1F1) or pituitary-specific positive transcription factor 1; TPIT, T-box transcription factor 19 (TBX19).

**Figure 2.**
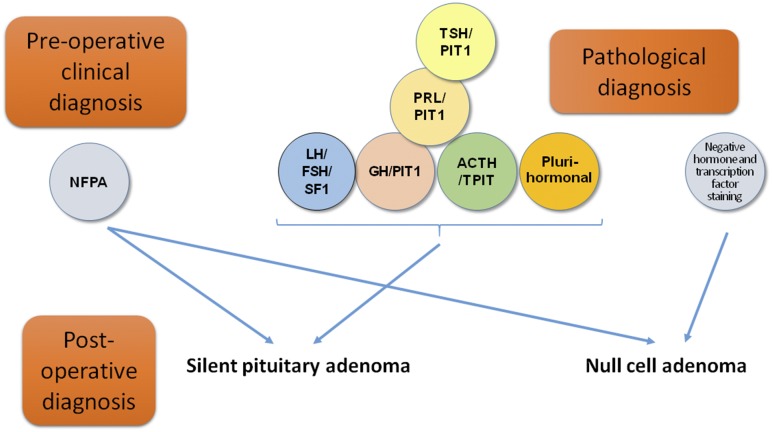
Silent pituitary adenoma is a diagnosis that can be determined by combining the clinical data (clinically nonfunctioning adenoma) and histopathological data (immunostaining for hormones and transcription factors).

Pituitary adenomas, more recently referred to as pituitary neuroendocrine tumors in line with neuroendocrine tumors (PitNets) from other organs (1), are common neoplasms comprising ~10% to 20% of intracranial tumors (2). The 2017 World Health Organization classification for endocrine tumors (3) now defines pituitary adenomas according to their pituitary hormone and transcription factor profile (Fig. 1; Table 1) (4). A sizeable proportion of pituitary adenomas (22% to 54% in different series) will present with signs and symptoms of mass effect rather than excessive hormone secretion and are defined as clinically nonfunctioning pituitary adenomas (NFPAs) (5–8). The term “silent pituitary adenoma” (SPA) refers to tumors that express one or more anterior pituitary hormones or their transcription factors with immunohistochemistry (IHC) but do not secrete hormones at a clinically relevant level (9, 10). The definition of null cell adenoma is restricted to an exceedingly rare primary adenohypophyseal tumor that is hormone negative with IHC and does not express any of the pituitary transcription factors. Therefore, the diagnosis of a clinically defined NFPA could be converted to an SPA if one were to consider the clinical findings of a nonfunctioning lesion and the pathological features showing positive hormone or transcription factor staining (Fig. 2). The present review has focused on SPAs as defined in this paragraph.

**Table 1.** Classification of Silent Pituitary Adenomas According to Adenohypophyseal Hormones and Transcription Factors

Cell Lineage	Pituitary Hormones by IHC	Transcription Factors and Other Cofactors
Somatotroph adenoma	GH, *α*-subunit	PIT1
Lactotroph adenomas	PRL	PIT1, ER*α*
Thyrotroph adenomas	TSH*β*, *α*-subunit	PIT1, GATA2
Corticotroph adenomas	ACTH	TPIT
Gonadotroph adenomas	FSH*β*, LH*β*, *α*-subunit	SF1, GATA2, ER*α*
Null cell adenomas	None	None
PIT1-positive adenomas	GH, PRL, TSH*β*, *α*-subunit	PIT1

Derived from data from Mete O, Lopes MB. Overview of the 2017 WHO Classification of Pituitary Tumors. Endocr Pathol 2017; 28:228-243.

**Figure 3.**
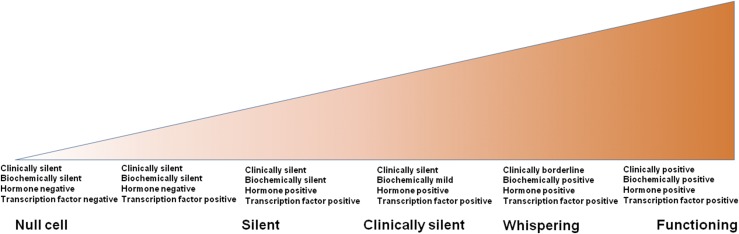
Continuous spectrum between silent and functioning adenomas.

The clinical picture of pituitary adenomas reflects a continuum between functional adenomas and “totally silent” adenomas (Fig. 3) (11). The phrase “totally silent” adenoma has been proposed for when the basal and stimulated serum concentrations of the corresponding hormones do not suggest excess hormone secretion and no clinical signs or symptoms are present that can be attributed to hormone excess (12). The phrase “clinically silent” can be used when SPAs secrete hormonal products that cause a mild elevation of the serum concentration but do not result in clinical signs or symptoms of hormonal hypersecretion (12). Some cases have been referred to as “whispering” adenomas with borderline, mild, often overlooked, clinical symptoms and signs but elevated hormone levels in the blood (11, 13). Furthermore, the functional status of a tumor can change during the course of the disease, which has most often been seen with ACTH-expressing tumors (14–16).

A comprehensive literature search was performed using MEDLINE and EMBASE databases from January 2000 to March 2018 with the following key words: (1) pituitary adenoma/tumor and nonfunctioning; or (2) pituitary adenoma/tumor and silent. Two separate searches were performed in each database without language restrictions. A total of 667 studies were retrieved (PubMed, n = 511; EMBASE, n = 156). Duplicate reports were excluded (n = 55). All titles and abstracts of the retrieved articles were reviewed, and the data are presented with an emphasis on the particular aspects of the different histological subtypes.

## General Characteristics

All types and subtypes of hormonally active adenomas can have a silent counterpart. Clinically presenting NFPAs are typically, although not always, macroadenomas, and patients frequently present with symptoms related to mass effects, such as headache, visual disorders, and/or cranial nerve dysfunction (17, 18). These tumors can also come to medical attention as an incidental finding when MRI is performed for unrelated signs and symptoms (19) or, less frequently, as a consequence of anterior pituitary hormonal deficiencies or hyperprolactinemia due to pituitary stalk compression (18). Guidelines for the management of incidental NFPAs have recently been published (20). NFPAs can also present as sinonasal or nasopharyngeal tumors without contact with the sella. In such cases, they should be differentiated from other tumor types occurring in this region such as primary or metastatic neuroendocrine tumors or olfactory neuroblastomas (21).

The prevalence of different histological types in a large surgical series of 1071 pituitary adenomas, including 555 functioning adenomas and 516 SPAs, has been summarized in Table 2 (10). Additionally, Nishioka *et al.* (10) showed the value of using a broad panel of cell lineage transcription factors to further classify hormone-negative adenomas into the exact type and subtype.

**Table 2.** Prevalence of SPAs Among Surgically Resected Pituitary Tumors

Histologic Type	Percentage of All Pituitary Tumors (IHC for Pituitary Hormones)	Percentage of All Pituitary Tumors (IHC for Pituitary Hormones and Transcription Factors)	Percentage of Silent Tumors Within Histologic Subtype (IHC for Pituitary Hormones)	Percentage of Silent Tumors Within Histologic Subtype (IHC for Pituitary Hormones and Transcription Factors)	Difference in Invasiveness and Recurrence Compared With Silent Gonadotroph Adenomas
Gonadotroph	28.2	35.6	99.3	99.5	Not applicable
Null cell adenoma	11.1	0.6	100	100	More invasive; greater surgical reintervention rates
GH/PRL/TSH lineage	46.2	48.4	9.2	9.7	Larger tumor size
					Greater recurrence rates
Corticotroph	14.5	17.5	32.9	44.4	Frequently giant adenomas; marked cavernous sinus invasion; greater recurrence rates

Derived from data from Nishioka H, Inoshita N, Mete O, et al. The complementary role of transcription factors in the accurate diagnosis of clinically nonfunctioning pituitary adenomas. Endocr Pathol 2015; 26:349-355.

The question to be addressed is whether and how the management of SPAs would change with an accurate pathological diagnosis. To date, few studies have reported treatment outcomes with consideration of the full pituitary hormone and transcription factor profile, because IHC staining for transcription factors regulating pituitary development is not yet widely available and most pathology laboratories still rely solely on pituitary hormone staining and, often, an incomplete panel. Nevertheless, the definition of pituitary adenomas according to their pituitary hormone and transcription factor profiles could potentially aid in predicting the disease course and response to adjunctive therapies.

## Null Cell Adenomas and Silent Gonadotroph Adenomas

**Figure 4.**
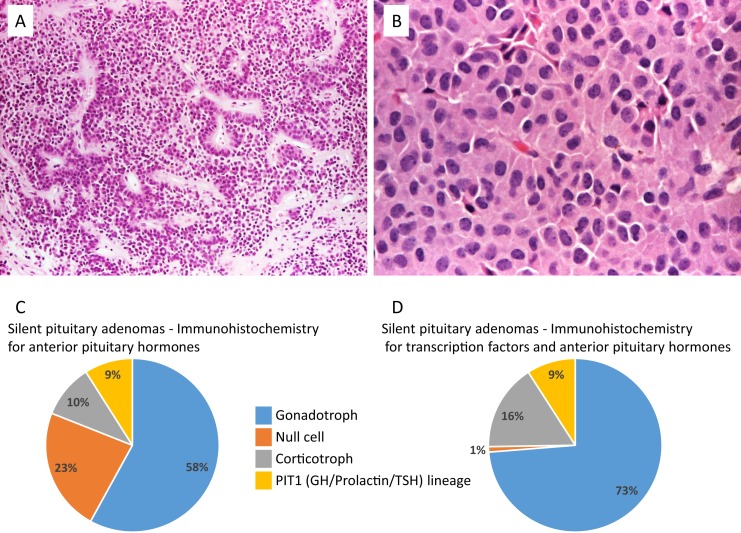
True null cell adenomas are composed of uniform, mildly atypical cells with chromophobic cytoplasm. (A) An example showing sinusoidal, papillary, and pseudopapillary architecture similar to the more common gonadotroph adenomas (hematoxylin and eosin stain, ×10). (B) A case of oncocytoma consisting of large cells with acidophilic, granular cytoplasm (hematoxylin and eosin stain, ×20). (C and D) Prevalence of the different SPAs subtypes according to IHC for anterior pituitary hormones: silent gonadotroph adenomas were the most frequent, followed by null cell (according to the World Health Organization 2004 classification), corticotroph, and GH/prolactin/TSH lineage adenomas. The new classification using IHC for anterior pituitary hormones and transcription factor profiling substantially reduced the number of null cell adenomas. Derived from data from Nishioka H, Inoshita N, Mete O, et al. The complementary role of transcription factors in the accurate diagnosis of clinically nonfunctioning pituitary adenomas. Endocr Pathol 2015; 26:349-355.

Unlike previous classifications (22), the 2017 World Health Organization “Blue Book” differentiates between null cell adenomas and silent gonadotroph adenomas (SGAs). Null cell adenoma is a diagnosis of exclusion that requires immunonegativity for all adenohypophyseal hormones and a lack of cell type-specific transcription factors (23). Null cell adenomas can also show oncocytic changes, characterized by large cells with cytoplasm densely filled with coalescent eosinophilic granules on light microscopy, which correspond, ultrastructurally, to numerous mitochondria (Fig. 4A and 4B) (24). This histopathologic finding has been suggested by some to be an indication of an aging pituitary neuroendocrine tumor and has not been associated with more aggressive behavior in a cohort of pituitary adenomas, including hormone-negative ones (25).

The introduction of steroidogenic factor 1 (SF1; coded by the nuclear receptor subfamily 5 group A, member 1 gene) IHC in the diagnosis of pituitary lesions has shown that many hormone-negative adenomas are, in fact, SGAs (26). It has been questioned whether null cell adenomas really exist or if this diagnostic category is merely a result of methodological diagnostic limitations (27). The expression of DAX1 (dosage-sensitive sex reversal, adrenal hypoplasia critical region, on chromosome X, gene 1), a member of the nuclear receptor superfamily involved with pituitary gonadotroph differentiation, was also studied, showing expression in clinically defined NFPAs, even with absent immunostaining for pituitary hormones, *α*-subunit, and SF1 (28).

The preoperative differential diagnosis of NFPAs includes, in addition to silent pituitary adenomas, primary non–hormone-secreting lesions such as craniopharyngioma, sellar paraganglioma, sellar neuroblastoma, sellar neurocytoma, metastasis from low-grade neuroendocrine tumor, lymphoma, and primary tumors of the pituitary stalk (29). The diagnostic immunopanel should therefore be expanded to include cytokeratins, S-100 protein (soluble in saturated 100% ammonium sulfate solution, a marker of schwannian, melanocytic, or chondrocytic tumors), and thyroid transcription factor-1 (a marker of lung and thyroid neoplasms). Correlation with the clinical history and neuroimaging findings is also mandatory (30).

The distinction of null cell adenomas and SGAs is of clinical relevance because null cell adenomas are likely to be more invasive than are SPAs (31). In a retrospective series of 516 patients with SPAs, 23.1% of the SPAs were initially classified as null cell adenomas using pituitary hormone IHC. However, IHC for PIT1 (pituitary-specific positive transcription factor 1; coded by the POU class 1 homeobox 1 gene), SF1, TPIT (coded by the T-box transcription factor 19 gene), and estrogen receptor-*α* (ER*α*) allowed for the identification of mutually exclusive lineage-specific markers in 95% of the cases, reducing the prevalence of the null cell adenoma subtype to ~1% (10) (Fig. 4C and 4D). The remaining genuine null cell adenomas proved to have an unfavorable outcome compared with hormone-negative but transcription factor–positive adenomas. In another study, in which the classification was also based on both adenohypophyseal hormone and PIT1 and SF1 immunochemistry (TPIT was not studied), SGAs represented 74.4% of the SPAs (32). The absence of p27 (cyclin-dependent kinase inhibitor 1B) expression was suggested to be prognostic for null cell adenomas, with early recurrence, cavernous sinus invasion, and a Ki-67 labeling index being higher in null cell adenomas compared to SGAs (31).

Although the vast majority of gonadotroph adenomas come to medical attention as clinically defined NFPAs, gonadotrophin secretion, primarily FSH secretion, has been shown in a high proportion of cases both *in vivo* and *in vitro*. In a cohort of 38 patients with SPAs, consisting of hormone-negative and gonadotroph adenomas, preoperative serum showed a median LH/FSH ratio of 0.33:1, with 35 of 38 patients presenting with LH/FSH ratios <1.0. Preferential secretion of FSH was also observed in *in vitro* culture media, with a median LH/FSH ratio similar to the preoperative serum ratio (0.32:1). In contrast, peritumorous “normal” pituitary cells secreted LH and FSH in an inverted ratio of 3.6:1. These data suggest, therefore, that a high percentage of these tumors release FSH into the circulation (33). Even lower serum LH/FSH ratios have been found in clinically functioning gonadotroph adenomas compared with SGAs (34).

In a retrospective series of 118 surgically resected gonadotroph adenomas without symptoms of hyperfunction, 48% of the men and 25% of the premenopausal women presented with elevated FSH and LH levels; isolated FSH elevation was more common (13%) than isolated LH elevation (8%) (35). In a clinicopathological analysis of 100 (79 men and 21 women) gonadotroph adenomas, hypogonadism was diagnosed in 78% of the men, and high levels of testosterone were not described. Hypersecretion of FSH and LH (defined as more than a twofold increase above the upper limit of normal) was observed in 19% and 9% of the men, respectively. In contrast, preoperative FSH and LH were not elevated in any of the premenopausal or postmenopausal women. Also, 33% of the patients presented with hyperprolactinemia, which might have altered the serum gonadotropin levels (36).

Circulating LH and FSH levels can therefore aid in the preoperative diagnosis and the postoperative surveillance of these patients. In men, a large sellar mass associated with elevated FSH, an inappropriately normal LH, and a low testosterone level or an elevated LH level, with or without elevated FSH, and a high testosterone level, is indicative of a gonadotroph adenoma (12). In premenopausal women, a gonadotroph adenoma should be suspected when elevated FSH, low or normal LH, pronounced elevated estradiol are present with clinical findings resembling polycystic ovarian syndrome, characterized by large polycystic ovaries and menstrual irregularities (37, 38). In postmenopausal women, the diagnosis can be more challenging because they typically have high circulating gonadotrophins with higher FSH than LH levels. However, the finding of elevated FSH with low or low-to-normal LH associated with a very large sellar mass should suggest the possibility of a gonadotroph adenoma in this patient group. Measurement of the *α*-subunit could also contribute to a preoperative diagnosis in clinically silent (but biochemically secreting) SPAs, because it might be the sole biochemical marker of the gonadotroph subtype in a number of cases (39).

The role of ER*α* has emerged as a prognostic factor in male patients with SPAs. Low expression is related to an earlier and greater repeat intervention rate in male patients with SGAs. Furthermore, in male patients with SGA, the combination of the absence of ER*α* expression and young age served as good predictive marker for repeat intervention (32). Androgen receptors are also often expressed in SGAs (40); however, their significance is unclear. Because SGAs and hormone-negative pituitary adenomas represent the most prevalent SPA subtypes, considerably more data are available on treatment strategies compared with most other types. Dopamine receptor type 2 (D2R) and somatostatin receptor (SSTR) expression have been demonstrated in both gonadotroph and hormone-negative adenomas, prompting the investigation of dopamine agonists and somatostatin analogs as potential treatment strategies (41–43).

D2R mRNA expression was demonstrated in two-thirds (12 of 18) of patients with an adenoma immunonegative for ACTH, GH, prolactin (PRL), and TSH (44). The nine patients presenting with residual tumor were treated with cabergoline, ≤3 mg/wk. After 12 months of treatment, tumor shrinkage was observed in 56% of the patients, and tumor reduction was significantly greater for those showing D2R expression (44). In another study of 9 patients with SGAs (immunonegative for GH, ACTH, PRL, and TSH and positive for LH, FSH, and/or *α*-subunit and >50% cells expressing D2R) with postoperative residual tumor present, 3 mg/week of cabergoline caused a >25% tumor volume reduction in six of the nine patients after 6 months (45).

Two reference centers in Israel have recently reported historical cohort analyses on the adjunctive role of a dopamine agonist in adult patients with GH- and ACTH-negative SPAs (46). The treatment group consisted of either patients who had started dopamine agonist therapy on detection of postoperative residual tumor (n = 55) or those who presented with tumor progression during follow up (n = 24). The control group (n = 60) received no medication after surgery. The dopamine agonist dose was aimed at 10 mg of bromocriptine daily or 2 mg/week of cabergoline, with a mean follow-up period of 8.8 ± 6.5 years. Tumor control, defined as tumor shrinkage or stabilization, was achieved in 87.3% of the patients who received treatment on detection of postoperative residual tumor, in 58.4% of those who received treatment after presenting with tumor progression, and in 46.7% of the control group. The requirement for additional surgery and radiotherapy during the follow-up period decreased from 46.7% to 16.4% with preventive treatment. No correlation was found between the clinical response to the dopamine agonist and D2R expression.

These encouraging results have initiated randomized controlled trials assessing the effects of cabergoline on NFPAs currently in progress. A phase III randomized controlled trial (ClinicalTrials.gov identifier, NCT03271918) assessing the use of cabergoline in patients with ACTH-negative SPAs who were not cured by surgery has recently been completed, and the results should be available shortly. In addition, a phase III randomized controlled trial (ClinicalTrials.gov identifier, NCT02288962) evaluating the effects of cabergoline on the change in tumor volume, both as primary treatment and as an adjuvant treatment of postoperative residual or progressive disease, is ongoing.

Initial studies using the SSTR2 agonist octreotide showed no or little effect on the tumor growth of clinically defined NFPAs (47, 48). More recently, a case-control study evaluated the results of long-acting octreotide in a cohort of 39 patients with heterogeneous SPAs (76.5% corresponding to the gonadotroph or hormone-negative types) presenting with a postoperative residue. The results demonstrated stabilization of the tumor remnant in 21 of 26 patients (81%) in the treated group compared with 6 of 13 patients (47%) in the control group, after a mean follow-up period of 37 months. However, neither visual field nor pituitary function was significantly changed in either of the groups, and no evidence of tumor shrinkage was found in any of the treated or control patients (49). The limited observed effect of octreotide on tumor shrinkage in that study could be due to SSTR5 being the predominant SSTR expressed (84%), followed by SSTR3 (61%), while SSTR2 was expressed in only 46% of the cases. SSTR2 expression has been also shown to be low in SPAs with immunonegative staining for all pituitary hormones or positive only for glycoprotein hormones (LH/FSH) compared with active somatotroph adenomas (42).

The expression of SSTRs and zinc finger protein regulator of apoptosis and cell-cycle arrest (ZAC1), a factor associated with the response to somatostatin analog therapy in patients with acromegaly (50), was assessed using quantitative RT-PCR (qRT-PCR) in 20 clinical NFPAs (18 SGAs and 2 hormone-negative adenomas) and compared with 23 active somatotroph adenomas and 5 normal pituitaries (51). The expression of SSTR2 and ZAC1 was decreased and SSTR3 expression was increased in the hormone-negative tumors and SGAs compared with the active somatotroph adenomas and normal pituitary (51). Likewise, other studies have suggested that SSTR3 is the predominant SSTR expressed in hormone-negative adenomas and SGAs, both by IHC studies and mRNA levels (32, 43, 52), and SSTR2 expression was shown to be absent in another cohort of true null cell adenomas (32). Some other studies, however, have reported greater SSTR2 expression than SSTR3 or SSTR5 in SGAs and hormone-negative adenomas (53, 54).

An ongoing phase II randomized controlled trial is evaluating pasireotide, a multiligand somatostatin analog with an action on SSTR1, SSTR2, SSTR3, and STR5 subtypes (55) in the treatment of clinically defined NFPAs. PASSION-1 (ClinicalTrials.gov identifier, NCT01283542; evaluate the efficacy and safety of pasireotide LAR on the treatment of patients with clinically nonfunctioning pituitary adenoma) is evaluating long-acting pasireotide as primary treatment of asymptomatic patients with clinically nonfunctioning macroadenomas. Additionally, the results of a phase II randomized controlled trial (ClinicalTrials.gov identifier, NCT 01620138) comparing the effects of cabergoline vs pasireotide in patients with various histological types of SPAs who were not cured by surgery are expected shortly.

Immune checkpoint inhibitors are widely used in the treatment of several types of cancers, and damage to the pituitary gland is one of the well-described side effects. However, one question is whether they could be used therapeutically for pituitary adenomas. Cytotoxic T-lymphocyte antigen 4, programmed cell death 1, and its ligand, programmed cell death ligand 1, are intrinsic downregulators of immunity and are often overexpressed in the tumor microenvironment (56). Programmed cell death ligand 1 mRNA and protein expression and lymphocytic infiltrate were greater in functioning pituitary adenomas than in clinically nonfunctioning adenomas (hormone-negative adenomas and SGAs) (57), providing a theoretical rationale for checkpoint inhibitor treatment of these tumors.

## Silent Somatotroph Adenomas

Silent somatotroph adenomas are GH-immunoreactive tumors that lack clinical and biological signs of acromegaly. They represent ~2% to 4% of all pituitary adenomas in surgical series (58, 59). Although patients with truly silent somatotroph adenomas will have normal preoperative GH and IGF-1 levels (58, 60), other cases will be clinically apparently silent but will show nonsuppressible serum GH and elevated IGF-1 levels (whispering adenomas) (61–64). In some cases, *in vitro* GH secretion with a positive response to GHRH stimulation has been observed (65). Clinically silent somatotroph adenomas have also been described in patients with aryl hydrocarbon receptor-interacting protein mutations, a tumor suppressor gene associated with familial isolated pituitary adenomas (66). IGF-1 (together with other biochemical parameters of pituitary function) should be measured preoperatively in patients with clinically defined NFPAs (67).

Silent somatotroph adenomas usually express less GH than their secreting counterparts (58); however, the mechanism associated with their reduced GH secretory capacity has yet to be clarified (67). In cases with very low positivity, the number of hormone positive cells above which we define positive immunostaining is not clear and thresholds >1% or >5% have been used. Moreover, immunostaining for transcription factors is especially valuable in such cases because it assists in the challenging differential diagnosis between scarcely positive immunostaining and normal entrapped pituitary cells staining for GH/PRL (59).

Secreting somatotroph adenomas are subclassified into densely granulated (DGSAs) and sparsely granulated (SGSAs). DGSAs show diffuse and strong positivity for GH and *α*-subunit and low-molecular-weight keratin stains in a perinuclear pattern. In contrast, SGSAs usually show focal or weak GH expression, no *α*-subunit expression, and substantial juxtanuclear globular reactivity for low-molecular-weight keratin (fibrous bodies). In addition, when a DGSA presents with scattered fibrous bodies, it can be referred to as an intermediate type somatotroph adenoma, with its biological outcome comparable to that of the densely granulated subtype (30). It is important to differentiate between these two subtypes because secreting SGSAs are usually more aggressive and might not respond well to somatostatin analog therapy (26).

Similar to functioning somatotroph adenomas, silent somatotroph adenomas can also be classified into DGSAs and SGSAs. Silent somatotroph adenomas are more frequently sparsely granulated than are active somatotroph adenomas (58). In a retrospective study that compared 21 silent and 59 secreting somatotroph adenomas, 85.7% of the silent somatotroph adenomas were SGSAs compared with 45.7% of their clinically active counterparts, and 95% were macroadenomas with the patients referred for symptoms of mass effects. The IGF-1 levels were within the normal range. In addition, compared with clinically functioning somatotroph adenomas, silent somatotroph adenomas were more common in women, presented with a lower percentage of GH immunoreactive cells, and were more frequently plurihormonal (GH/PRL or GH/PRL/TSH). No differences in the marker of proliferation, Ki-67 labeling index, tumor suppressor p53 expression, or prognostic grades [using the Trouillas classification (68)] were observed between silent somatotroph adenomas and secreting somatotroph adenomas.

Similar results were shown in a previous retrospective study, in which silent somatotroph adenomas accounted for 7 of 620 (1%) surgically removed pituitary tumors over 14 years (69). Despite the small sample number, the investigators concluded that, compared with secreting somatotroph adenomas, silent somatotroph adenomas were more frequent in females, presented at a younger age, and were larger, more invasive, and recurred earlier and more frequently.

In a recent single-center retrospective surgical series of pituitary adenomas, silent somatotroph adenomas represented 2% of the cases (60), and the prevalence of silent corticotroph adenomas (SCAs; discussed in more detail in the next section) and SGAs was 4.5% and 18.9%, respectively. In their cohort, silent somatotroph adenomas were smaller than the SGAs, presented at a younger age, and showed a substantial female preponderance. On histopathological analysis, they expressed GH, with 53% of cases coexpressing PRL. After a mean follow-up of 3.9 years, nearly one-third of the patients with silent somatotroph adenomas had experienced tumor progression or recurrence, and the rates of surgical repeat intervention or an indication for adjunctive radiation therapy were similar to those for SCAs but significantly greater than those for SGAs. Transition to acromegaly was observed in 2 of 17 cases (two female patients developed progression with elevated IGF-1 levels during the follow-up period). The clinical and pathological characteristics of pure silent GH adenomas were compared with those of the mixed GH/PRL tumors. Although those findings were limited by the small sample size, tumors with a pure GH immunoprofile and, more specifically, the sparsely granulated subtype, were likely to be larger and more invasive and showed greater recurrences rates.

The expression of SSTR2 and SSTR5 was recently demonstrated in all the silent somatotroph adenomas reviewed (n = 21); however, expression of SSTR2 was significantly lower compared with the secreting counterpart, and SSTR5 expression was similar in both groups (58). In another series, the expression of SSTR2 was observed in 6 of 11 cases (>50%); however, SSTR5 expression was not analyzed (60). Clinical data regarding the therapeutic effect of somatostatin analogs on silent somatotroph adenomas are not currently available.

## Silent Corticotroph Adenomas

Similar to other pituitary tumor types, corticotropinomas can be either functioning or nonfunctioning (3). SCA was the first silent adenoma subtype described as a distinct clinicopathologic entity (70). SCAs are characterized by the absence of the clinical features of Cushing syndrome and normal cortisol dynamics (totally silent) or elevated ACTH/abnormal cortisol dynamics (clinically silent) (6, 15, 71, 72). Crooke hyaline changes (*i.e.,* accumulation of perinuclear cytokeratin filaments in normal or neoplastic corticotrophs resulting in a glassy hyaline appearance on hematoxylin and eosin stains) will be absent in the normal part of the pituitary owing to the lack of cortisol excess (4, 73).

SCAs account for 3% to 6% of all pituitary adenomas, 10% to 20% of SPAs, and ~40% of all corticotroph cell tumors (6, 15, 74–76). There is a spectrum of functionality of SCAs, and it has been questioned whether they are truly silent. Acquired postoperative adrenal insufficiency has been reported in 20% to 30% of patients with adenomas that are apparently SCAs (60), perhaps indicating that these tumors actually secrete enough ACTH locally to suppress ACTH secretion by normal corticotrophs (72). A recent, prospective, well-designed study addressed this question. It evaluated the hypothalamic–pituitary–adrenal axis after transsphenoidal surgery in patients with SCAs compared with patients with ACTH-negative SPAs. They included patients in whom neither cortisol deficiency nor cortisol excess was noted in their preoperative assessment (a normal plasma ACTH concentration and a random serum cortisol level of ≥12 μg/dL was used to assess ACTH deficiency; however, dynamic tests such as dexamethasone suppression or midnight cortisol were not assessed) (74). During perioperative stress, the pattern and extent of hypothalamic–pituitary–adrenal axis activation in patients with SCAs was not different from that in those with ACTH-negative SPAs, suggesting that these tumors were truly nonfunctional.

**Figure 5.**
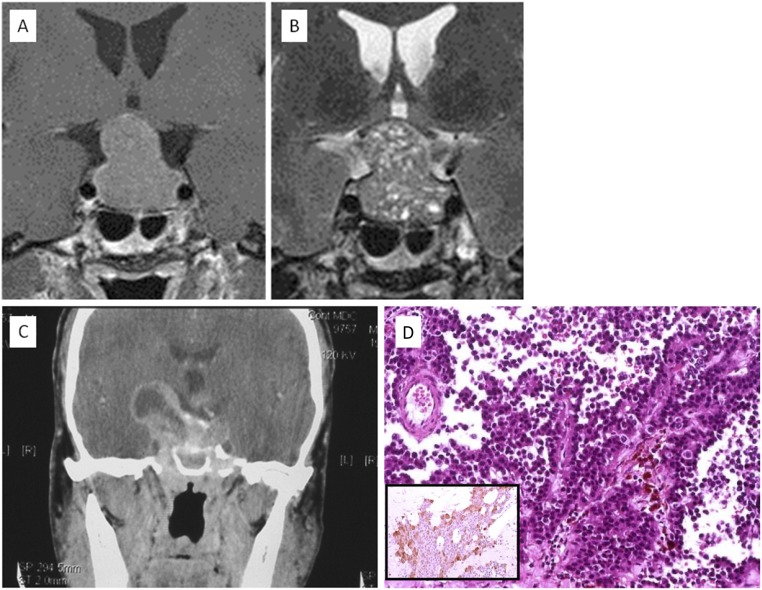
Pituitary MRI scans of a silent corticotroph adenoma. (A) T1-weighted MRI sequence and (B) T2-weighted MRI sequence allowing identification of multiple microcysts. [Figure kindly provided by Prof. Bonneville, Lyon, France (130)]. Extension to parasellar structures, invasion of the sellar floor and cystic and hemorrhagic changes can be features of silent corticotroph adenomas. This example of a cystic silent ACTH adenoma type 2 adenoma had invaded both clivus and clinoids and extended superiorly to the third ventricle. (C) Axial contrast-enhanced CT scan). (D) Histologically, the lesion had papillary architecture (hematoxylin and eosin stain, ×20). (Inset) A few neoplastic cells expressed ACTH (immunoperoxidase stain, ×20). (A and B) Derived from data from Bonneville F. Silent corticotroph pituitary adenoma. In: Bonneville JF, Bonneville F, Cattin F, Naggi S, eds. MRI of the Pituitary Gland. Springer; 2016.

SCAs usually present as macroadenomas associated with mass-related symptoms (76). Patients with SCAs are younger than patients with SGAs and true null cell adenomas (32). In contrast to SGAs, SCAs show a female preponderance, are more frequently giant adenomas, and are more often associated with marked cavernous sinus invasion (10). The presence of cystic and hemorrhagic components in T2-weighted pituitary MRI sequences in a NFPA might point toward the corticotroph subtype (77). In this recent retrospective series, cystic components, including both macro- and microcysts, were observed on T2-weighted MRI scans in all patients with SCAs (77). In contrast, the specific finding of multiple microcysts was present in 76% of patients with SCAs vs only 5% of patients with SGAs (Fig. 5A and 5B). The presence of multiple microcysts in clinically silent pituitary macroadenomas had a sensitivity of 76% and a specificity of 95% for predicting an SCA (77). A correlation between these MRI findings and pseudopapillary features on pathological examination was also demonstrated in patients with SCAs. All the tumors with pseudopapillary features presented with multiple microcysts on T2-weighted MRI sequences. The few patients whose T2-weighted MRI sequences shown no multiple microcysts had no pseudopapillary features on pathological examination of the SCA (77).

Corticotroph adenomas can be divided into two subtypes: type 1 SCA (densely granulated) and type 2 SCA (sparsely granulated) (6, 10, 30). Type 1 SCAs are indistinguishable from Cushing-related microadenomas and show strong ACTH immunoreactivity. Type 2 SCAs resemble the rare chromophobe corticotroph adenoma and show weak and focal ACTH immunoreactivity (Fig. 5C and 5D). In such cases, positive immunostaining for low-molecular-weight cytokeratin and TPIT will help to confirm the diagnosis (78). To refine the differential diagnosis between functioning and silent corticotropinomas, Thodou *et al.* (79) proposed the assessment of galectin-3. In their experience, >80% of SCAs lacked galectin-3 expression, and galectin-3 was uniformly present in hormonally active adenomas, including a case of Crooke cell adenoma, a rare and reportedly aggressive variant of corticotroph adenoma showing Crooke hyaline changes in >50% of neoplastic cells. Silent Crooke cell adenomas have been described in both adults and children (80, 81). Type 2 SCAs seem to be more common than type 1 SCAs (9) and have a tendency to display greater expression of factors that regulate cell invasion, migration, and proliferation, such as fibroblast growth factor receptor-4, matrix metalloproteinase-1, and *β*1-integrin, compared with type 1 SCAs (82).

**Figure 6.**
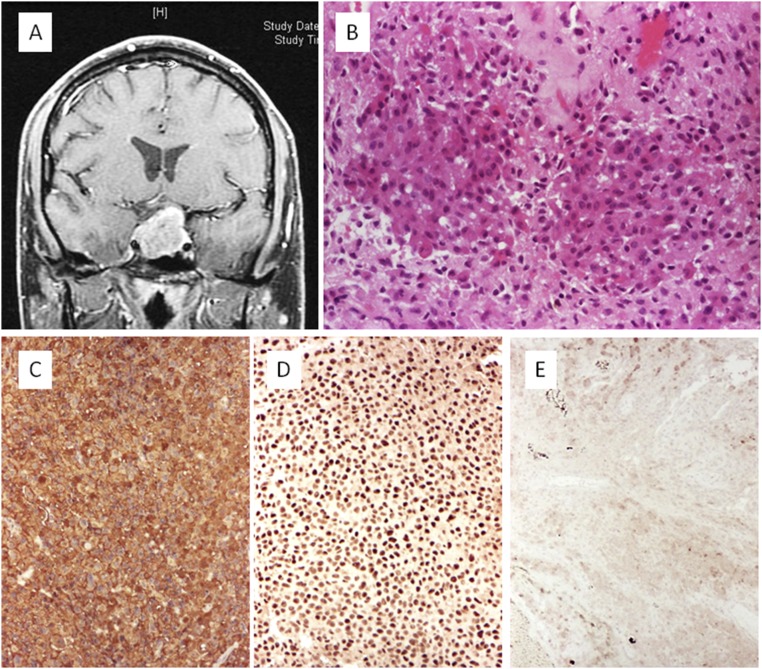
Silent corticotroph adenoma: noninvasive macroadenoma. (A) Axial contrast-enhanced T1-weighted sequence. (B) Histologic slide showing sheets and acini with uniform, medium-size cells with basophilic cytoplasm (hematoxylin and eosin stain, ×20). (C) Expression of ACTH is diffuse (immunoperoxidase stain, ×10). (D) Neoplastic cells show nuclear expression of the transcription factor TPIT (immunoperoxidase stain, ×10). (E) No expression of PC1/3 was present in the tumor cells (immunoperoxidase stain, ×10).

The question remaining is why a tumor that synthesizes ACTH does not cause the associated clinical syndrome. One of the theories that has been proposed is that the cells originating functioning ACTH-secreting adenomas or SCAs have different locations within the pituitary gland. Those associated with Cushing disease are suggested to arise from the ACTH-positive cells in the anterior pituitary, and SCAs might arise from the proopiomelanocortin (POMC)-producing cells in the pars intermedia, which, in turn, demonstrates a low ACTH secretory capacity (72, 83). The hypothesis that SCAs derive from an intermediate lobe cell that shares both gonadotroph and corticotroph characteristics is based on a previous demonstration by electron microscopy and immunopositivity for transcription factors consistent with the gonadotroph cell line in a cohort of 18 SCAs (84). However, this finding was not confirmed in a subsequent study (60) and requires further investigation. It has also been proposed that SCAs secrete predominantly high-molecular-weight ACTH, which could compete with the normal ACTH (1 to 39 amino acids) at the receptor level (85). Other suggested mechanisms include increased intracellular degradation of ACTH and failure of exocytosis of hormone from the cell membrane (70). An attractive, and currently most likely, hypothesis is that the clinical manifestations of Cushing disease are dependent on the processing of the prohormone POMC in corticotrophs. Prohormone convertase 1/3 (PC1/3) is involved in the post-translational processing of POMC into mature and biologically active ACTH (86). Studies have demonstrated a decrease in PC1/3 expression associated with a downregulation of PC1/3 genes in SCAs compared with corticotroph adenomas associated with Cushing disease (71, 87). The immunohistochemical features of a PC1/3-negative and ACTH-positive SCA are illustrated in Fig. 6. In patients with SCAs presenting with elevated mean preoperative plasma ACTH levels but normal serum cortisol levels, POMC expression, measured using qRT-PCR, was similar to that of corticotroph adenomas causing Cushing disease and several hundred times greater than that in hormone-negative adenomas. In contrast, the expression of PC1/3 was 30-fold higher in those with Cushing disease compared with that in those with SCAs (88). The investigators suggested that the elevated ACTH plasma levels observed in patients with SCAs could reflect increased circulating POMC detected by the ACTH plasma immunoassay.

**Figure 7.**
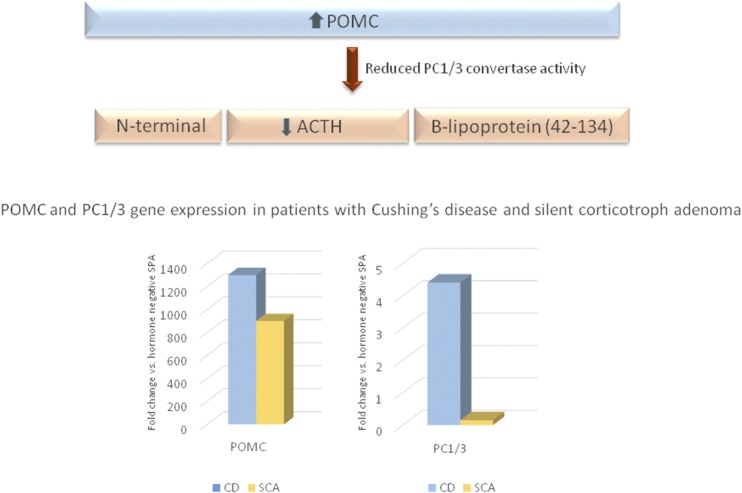
Proposed mechanism for “silencing” of corticotroph adenomas. Reduced PC1/3 activity decreases tumor cell production of ACTH despite increased levels of POMC. Compared with hormone-negative adenomas, POMC gene expression is increased in pituitary tumor samples from patients with Cushing disease (CD) and patients with SCAs; however, transcripts for PC1/3 are present at 30-fold greater levels in those with Cushing disease than in those with SCAs. Derived from data from Jahangiri A, Wagner JR, Pekmezci M, et al. A comprehensive long-term retrospective analysis of silent corticotrophic adenomas vs hormone-negative adenomas. Neurosurgery 2013;73:8-18.

Another intriguing question is how SCAs can evolve into Cushing disease after years of inactivity. Transformation of an SCA to Cushing disease was first reported in 1985, after an 18-year follow-up period for a patient who originally had a clinically defined NFPA (89). Recently, a change in the tumor phenotype, from SCA to Cushing disease, but also from Cushing disease to SCA, was reported in 3.9% of SCAs studied, with a transformation time of 1 to 7 years (15). The expression of PC1/3 was analyzed by immunochemistry and qRT-PCR in tissue specimens from both phases in three of the patients who had presented with transformation from a SCA to Cushing disease. PC1/3 expression was negative or weak in the three patients in the initial presenting phase of SCA, but robust expression was detected in tissue specimens acquired from the same patients at the detection of recurrence as Cushing disease (15). These findings underscore the role of PC1/3 as one of the main potential mechanisms for “silencing” corticotroph adenomas (Fig. 7) and might provide information on the mechanisms associated with the other SPA subtypes.

A recent retrospective series of SCAs was reviewed in the search for predictors of recurrence, comparing the findings to a cohort of patients with SGAs (60). The SCAs were of similar size and invasiveness compared with the SGAs but showed substantially greater recurrence rates (36% vs 10%), in conjunction with a higher number of patients with SCAs requiring adjuvant radiotherapy (18% vs 3%). In this series, none of the patients with recurrent SCAs had cystic tumors (defined as tumors with >50% fluid content based on the MRI T2 signal). The investigators concluded that fewer cystic tumors and greater preoperative ACTH levels were characteristics substantially associated with recurrence. Another large retrospective analysis compared 75 SCAs and 1726 adenomas with negative immunostaining for ACTH, PRL, and GH (LH/FSH staining was not reported) (88). The investigators were able to demonstrate that SCAs were more likely to exhibit cavernous sinus invasion and greater progression and/or recurrence rates after a mean follow-up of ~3 years (88).

In contrast to other SPAs, SCAs have demonstrated a shorter time to recurrence after transsphenoidal surgery (84) and a higher rate for adenoma progression and hypopituitarism after stereotactic radiosurgery (90). This latter retrospective multicenter study evaluated the effectiveness of stereotactic radiosurgery in 50 patients with SCAs and 307 patients with other SPA subtypes who had undergone at least one transsphenoidal surgery (90). The factors shown to affect tumor progression rates were the presence of ACTH staining and the margin dose. Therefore, it has been suggested that in SCAs, an elevated margin dose might be considered to achieve a better chance of tumor control.

Two recent retrospective series found no critical risk factors predicting recurrence after primary or secondary treatment of SCAs. A single-center retrospective study evaluated 108 surgically resected SPAs followed for ≤15 years (91). Of their patients, 22% required further treatment, either second surgery or radiotherapy (91). The factors determining recurrence were the presence of residual tumor, tumor growth rate (>80 mm^3^/y), and suprasellar extension. In contrast, the SPA type, categorized by anterior pituitary hormone immunostaining, was not a predictive factor for the requirement for secondary treatment. However, the percentage of SCAs within the studied SPAs was only 3%, limiting the interpretation of these results (91). In another retrospective study from two UK reference centers evaluating patients with SPAs (17% with positive staining for ACTH) who presented with tumor regrowth after primary treatment, the anterior pituitary hormone immunostaining profile of the adenoma was not a substantial factor for further tumor regrowth (92). The important risk factors were female sex and treatment modality; the incidence of secondary regrowth was greater in the conservative monitoring group (63%) than in the surgery (36%), radiotherapy (13%), or surgery/adjuvant radiotherapy (13%) groups.

The expression of SSTRs and D2R was evaluated in 8 SCAs using qRT-PCR and compared with 15 ACTH-negative SPAs and 12 corticotroph tumors associated with Cushing disease (93). The D2R mRNA levels were lower in the SCAs and secreting corticotroph adenomas than in the ACTH-negative SPAs. The SSTR1 mRNA levels were greater in the SCAs than in the two other groups, the SSTR2 levels were greater in the SCAs than in the secreting corticotroph adenomas, and SSTR3 levels were low and similar in all the groups. The SSTR4 levels were undetectable, and the SSTR5 levels were detectable, but lower, in the SCAs compared with the secreting corticotroph tumors (93). In another study, 15 SCAs demonstrated greater immunoreactive scores for SSTR2 compared with null cell adenomas (defined in that study as hormone-, SF1-, and PIT1-negative samples; n = 10) and greater immunoreactive scores for SSTR5 compared with SGAs (n = 110) (32). SSTR3 was expressed abundantly by all types of SPAs, including SCAs (32). The significance of these findings regarding the potential use of dopamine agonists and, in particular, somatostatin analogs, in the treatment strategy for SCAs has not yet been determined. At present, PASSILCORT (pasireotide LAR therapy of silent corticotroph pituitary tumors; ClinicalTrial.gov identifier, NCT02749227), a phase II randomized clinical trial, is ongoing, aiming to evaluate the results of long-acting pasireotide therapy for patients with SCAs who present with residual or recurrent tumors.

## Silent Thyrotroph Adenomas

Although increasingly recognized (94), thyrotroph adenomas remain rare (95). These tumors accounted for 3.5% of the operations for pituitary adenomas performed at a single center, and most of these tumors were clinically silent (96). The most relevant finding of this series was compared with another large retrospective series from Japan (97) (Table 3). In both series, >50% of the silent thyrotroph adenomas presented with extrasellar extension, and the most common symptoms were visual disturbance and headache. When patients with TSH-expressing adenomas present with hyperthyroidism, the symptoms are often milder than those in patients with primary hyperthyroidism (98).

**Table 3.** Comparison of Clinical Data From Secreting and Silent Thyrotroph Adenomas

Study	TSH-Expressing Pituitary Tumors, n	Study Period	Percentage of All Pituitary Tumors	Percentage of Silent Tumors	Percentage of Macroadenomas in Silent Group	Larger Tumor Size Compared With Thyrotropinomas	Difference in Invasiveness/Recurrence Compared With Thyrotropinomas
Kirkman *et al.* (96), 2014	32	2002–2012	3.5	75	88	No	No
Wang *et al.* (97), 2009	29	1975–2001	<2.4	31	100	Yes	No

Thyrotroph adenomas usually show variable TSH*β* and *α*-subunit expression on IHC (4). Nuclear expression of PIT1 and its coexpression with GATA-binding protein-2 has been described in several cases (30, 97, 99). The percentage of TSH*β*-expressing cells has been shown to vary widely, ranging from 1% to 90%, with significantly greater expression in patients with hyperthyroidism (96). Furthermore, 84% of cases in that series expressed other hormones, with no substantial differences between the secreting and silent subgroups. Also, no difference was found in the pre- and postoperative imaging findings, postoperative complications, or recurrence rate (96). One-third of the patients had developed a recurrence after a mean follow-up of 80 months; however, consistent markers of recurrence could not be identified, with no differences in the percentage of TSH-expressing cells or Ki-67 index shown (96).

Immunoreactivity for SSTR2 and SSTR5 has been shown to be positive in 89% and 78% of silent thyrotroph adenomas, respectively, a difference that was not significantly different from the hormonally active adenomas (97). TSH-secreting adenomas generally show excellent hormonal and tumor size response to somatostatin analog treatment (94, 100–104). Because silent thyrotropinomas also express SSTRs, somatostatin analog treatment after surgery when a tumor remnant is present could be a viable option, as seen in individual cases (105).

## Silent Lactotroph Adenomas

Clinically presenting silent lactotroph adenomas are rare. More frequently, positive PRL expression with IHC is encountered as a feature of a silent mixed somatotroph-lactotroph adenoma, a morphological variant of somatotroph adenomas (60). According to the German Registry of Pituitary Tumors, the prevalence of silent lactotroph adenomas among SPAs was 1.65%, with most belonging to the sparsely granulated subtype (9). A recent retrospective surgical series of SPAs showed an even lower prevalence of only 0.6% (92). However, approximately one-half of the microadenomas identified in autopsy studies have stained positive for PRL (106).

## PIT1-Positive Plurihormonal Adenomas

**Figure 8.**
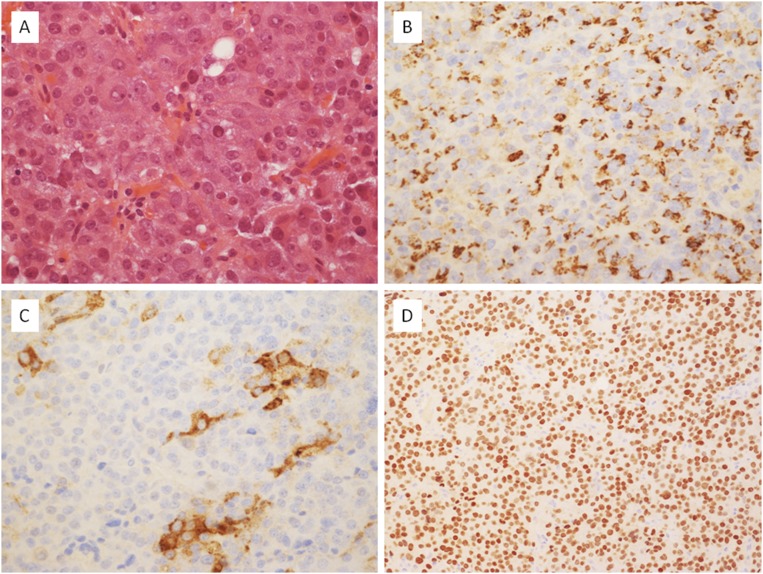
A case of a plurihormonal PIT1 adenoma showing the typical diffuse, solid architecture of this tumor type. (A) It was composed of epithelioid cells with eosinophilic cytoplasm, an enlarged nucleus, and prominent nucleolus (hematoxylin and eosin stain, ×40). (B) Some cells expressed PRL (immunoperoxidase stain, ×40). (C) A few tumor cells were positive for TSH *β*-subunit (immunoperoxidase stain, ×40). (D) Nuclear expression of the transcription factor PIT1 was ubiquitous (immunoperoxidase stain, ×20).

Formerly known as silent adenoma type III, PIT1-positive plurihormonal adenoma is defined as a plurihormonal lesion uniformly expressing PIT1 (3, 4). PIT1-positive plurihormonal adenomas are monomorphous; they usually express one or more hormones of the PIT1 lineage with only a small portion of them being hormone negative” (Fig. 8) (107).

PIT1-positive plurihormonal adenomas are a distinct entity, with reportedly aggressive behavior. A single-center retrospective series observed a prevalence of 0.9% for silent subtype 3 adenomas among resected pituitary tumors during a 13-year period. The classification was based on the ultrastructural features, histological aspects, and immunoreactivity for anterior pituitary hormones. All tumors were macroadenomas, 60% showed radiographic features of invasion, and the rate of persistent or recurrent disease was >50% during a median follow-up of 51 months (108). These tumors tend to occur in younger patients, are often not silent, and can present with clinical symptoms of hormonal excess. Thirty percent of the patients reviewed in their study presented with hormone hyperfunction, either GH excess (5 of 27) or substantial hyperprolactinemia (3 of 27), and 2 patients had a definite diagnosis of multiple endocrine neoplasia type 1 syndrome. In another retrospective series of 25 silent subtype 3 adenomas, substantial hormonal excess was also present in approximately one-third of the cases, including hyperthyroidism in 17%, acromegaly in 8%, and marked hyperprolactinemia in 4%. Association with the multiple endocrine neoplasia type 1 syndrome was again reported in this series in two of the patients who were younger than 30 years and presented with concomitant hyperprolactinemia and hyperparathyroidism (107).

## Aggressive SPAs

The clinical and pathological criteria defining aggressive adenomas have recently been proposed by a panel of experts (109). These criteria also apply to SPAs, which, rarely, can develop into metastatic tumors (110–114). Temozolomide was the first chemotherapeutic agent to demonstrate substantial response rates in aggressive pituitary tumors. Responsiveness to temozolomide is likely dependent on the immunoexpression of O (6)-methylguanine DNA methyltransferase (MGMT), a DNA repair protein that acts by removing the alkyl group and inducing resistance to temozolomide. Low immunoexpression of MGMT by pituitary tumors has been associated with high response rates to temozolomide (115).

To date, few case reports have concerned the use of temozolomide in patients who presented with aggressive SPAs or who developed pituitary carcinomas several years after the diagnosis of SPAs (116–119). MGMT immunoexpression was assessed in a group of 45 SPAs of various histological subtypes, and the degree of expression (low expression defined as ≤50% immunostained adenoma cells and high as >50%) correlated with tumor aggressiveness. Low MGMT expression was observed in 50% of the aggressive SPAs compared with 24% in the nonaggressive SPAs (120). Additionally, MGMT immunoreactivity was evaluated in 23 silent subtype 3 adenomas; 78% showed no MGMT immunoreactivity, 17% displayed immunoreactivity in <25% of the tumor cells, and none of the tumors showed high immunoreactivity (>50%) (121). These findings suggest that aggressive SPAs with low MGMT expression could be potential candidates for treatment with temozolomide (115); however, a recent survey performed by the European Society of Endocrinology showed that, in general, silent pituitary tumors were less likely to respond to temozolomide than secreting pituitary tumors, independent of MGMT expression status (109). Hormone-negative pituitary adenomas were more likely to display high MGMT expression (>50% positive cells) compared with hormone-positive tumors, irrespective of the functional status. Another interesting finding from that large cohort of aggressive pituitary tumors (n = 166) was the high proportion (26%) of initially silent corticotroph or somatotroph adenomas that evolved into clinically functioning tumors, again underscoring the continuous functional spectrum of pituitary adenomas (109).

## Future Prospects

The identification of cellular markers predicting tumor behavior is the “holy grail” of pituitary pathology. The role of cell proliferation and apoptosis markers in predicting the recurrence of SPAs has also been investigated. A high Ki-67 and TUNEL labeling indices and increased phosphorylated AKT (serine/threonine-specific protein kinase), phosphorylated MAPK (p44/42 MAPK), and PTTG1 (pituitary tumor-transforming 1) immunostaining were associated with early tumor recurrence (122). Also, high phosphorylated cyclic AMP response element-binding protein and ZAC1 expression correlated inversely with recurrence (122). In another study of 74 SPAs, including SGAs and hormone-negative adenomas, the Ki-67 index was substantially associated with a tumor size >3 cm and tumor recurrence, suggesting Ki-67 to be a consistent marker of biological behavior in these subtypes (53). The evaluation of tumor proliferation using Ki-67 immunochemistry is widely available and recommended as part of the assessment of SPAs (4).

A recently suggested biomarker for invasiveness of SPAs is ezrin (EZR) (123). *EZR* encodes ezrin, also known as villin-2 or cytovillin, a protein that serves as an intermediate between the plasma membrane and the actin cytoskeleton, in addition to being involved in the regulation of the growth and metastatic capacity of neoplastic cells (124). Invasive SPAs were shown to have significantly greater levels of *EZR* mRNA and of ezrin protein expression compared with noninvasive SPAs (125).

TGF-*β*/Smad signaling might be associated with the development and invasiveness of SPAs (126). TGF-*β* signaling is involved in a number of critical processes such as cell proliferation, differentiation, migration, apoptosis, and epithelial–mesenchymal transition (127). Low expression of TGF-*β* receptor type 2 might be related to the invasiveness of SPAs, because it has been shown that TGF-*β* receptor type 2 protein and mRNA levels were significantly lower in invasive SPAs compared with noninvasive SPAs and normal pituitary tissue (128). Furthermore, TGF-*β* receptor type 2 mRNA levels showed a negative correlation with proliferating cell nuclear antigen, a proliferative marker shown to be substantially greater in the invasive SPAs in this cohort (128). With the purpose of investigating further the TGF-*β*/Smad signaling role in SPA tumor development, Smad3 and phosphorylated Smad3 protein levels were measured by immunochemistry in 161 patients with SPAs, including 59 invasive (36.6%) and 102 noninvasive (63.4%) lesions (129). In agreement with previous findings, the protein levels of Smad3 and phosphorylated Smad3 were significantly lower in patients with invasive SPAs than in noninvasive SPAs, correlating inversely with the Ki-67 index.

## Conclusions

Silent pituitary adenomas represent a challenging diagnostic group of tumors. Close collaboration of the “pituitary team” is essential for a precise diagnosis and will contribute to the optimal treatment of the patient. New classifications, novel prognostics markers, and emerging imaging and therapeutic approaches will need to be evaluated to better serve this unique group of patients.

## Abbreviations:

D2R: dopamine receptor D2
DGSA: densely granulated somatotroph adenoma
ER*α*: estrogen receptor *α*
EZR: ezrin
IHC: immunohistochemistry
MGMT: O-6-methylguanine-DNA methyltransferase
NFPA: nonfunctioning pituitary adenoma
PC1/3: prohormone convertase 1/3
POMC: proopiomelanocortin
PRL: prolactin
qRT-PCR: quantitative RT-PCR
SCA: silent corticotroph adenoma
SF1: steroidogenic factor 1
SGA: silent gonadotroph adenoma
SGSA: sparsely granulated somatotroph adenoma
SPA: silent pituitary adenoma
SSTR: somatostatin receptor
ZAC1: zinc-finger protein regulator of apoptosis and cell-cycle arrest
